# To shock or not to shock?

**DOI:** 10.1007/s12471-025-01981-0

**Published:** 2025-09-02

**Authors:** Mónica Dias, Bárbara Antunes Rocha, Sérgia Rocha, Rui Files Flores

**Affiliations:** Department of Cardiology, Hospital of Braga, Braga, Portugal

## Question

A 70-year-old male patient with liver steatosis and prostatic carcinoma presented to the emergency department with pre-syncope and symptoms of congestive heart failure.

Initial evaluation revealed tachycardia, normal blood pressure, and signs of pulmonary congestion. Blood analyses showed elevated natriuretic peptides, with no other significant alterations.

**Fig. 1:**
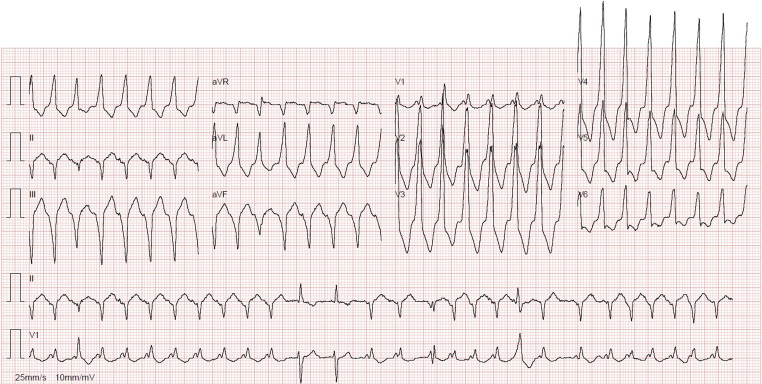
12-lead ECG. To shock or not to shock?

A 12-lead ECG was performed (Fig. 1). What is the diagnosis, and should we shock it or not?

## Answer

You will find the answer elsewhere in this issue.

